# Lamin-A/C Is Modulated by the Involvement of Histamine-Mediated Calcium/Calmodulin-Dependent Kinase II in Lung Cancer Cells

**DOI:** 10.3390/ijms23169075

**Published:** 2022-08-13

**Authors:** Hyeong-Jae Kim, Peter C. W. Lee, Jeong Hee Hong

**Affiliations:** 1 Department of Physiology, Lee Gil Ya Cancer and Diabetes Institute, College of Medicine, Gachon University, Incheon 21999, Korea; 2 Lung Cancer Research Center, University of Ulsan College of Medicine, Asan Medical Center, Seoul 05505, Korea; 3 Department of Biomedical Sciences, University of Ulsan College of Medicine, Asan Medical Center, Seoul 05505, Korea

**Keywords:** lamin-A/C, histamine signaling, calcium, Ca/calmodulin-dependent kinase II, lung cancer cells

## Abstract

Lamins are nuclear envelope proteins involved in various cellular functions, such as DNA modulation, cellular differentiation, and development. In this study, we investigate the role of histamine in lung cancer biology. Since it is known that lamin-A/C is negatively regulated in lung cancer, we hypothesize that histamine signaling is related to nuclear lamin-A/C regulation and cancer progression. Our findings reveal that histamine stimulation enhances lamin-A/C expression in lung cancer cells. Lamin-A/C expression is dependent on histamine-mediated intracellular calcium signaling and subsequent calcium/calmodulin-dependent kinase II (Ca/CaMKII) activation. The nuclear protein nestin, which stabilizes lamin-A/C expression, is also modulated by Ca/CaMKII. However, histamine-mediated lamin-A/C expression is independent of Akt/focal adhesion kinase or autophagy signaling. Histamine stimulation attenuates lung cancer motility in the presence of enhanced lamin-A/C expression. In conclusion, we propose a regulatory mechanism that accounts for the modulation of lamin-A/C levels through the involvement of Ca/CaMKII in cancer cells and provides molecular evidence of histamine signaling in lamin-A/C biology.

## 1. Introduction

Lamins are nuclear envelope-associated proteins which comprise two types: type A/C and type B. Lamins localize in the inner nuclear membrane and are involved in various cellular functions, such as the maintenance of nuclear shape, chromatin modulation, gene regulation, cellular differentiation, development, and aging-associated mechanisms [1,2,3,4,5,6,7,8]. Lamins are encoded by *LMNA* (type A, lamin-A and -C) and *LMNB* (type B, lamin-B1 and -B2), and mutations in lamin-A/C are associated with various human diseases, such as Emery–Dreifuss muscular dystrophy [9], limb-girdle muscular dystrophy [10], and Hutchinson–Gilford progeria syndrome [11], including cardiomyopathy [12] and osteoporosis [13]. Additionally, although >600 human mutations of *LMNA*, including slice variants, have been identified in the ClinVar database [14], the precise characterization of mutants or splice variants is required for future studies.

Although the roles of lamin-A/C have been developed in various fields, the direct modulation of lamin-A/C expression has been gradually discovered. Proteomic-based analysis of Akt for nuclear substrates indicates that lamin-A/C is a putative substrate of Akt [15]. Akt or Cdk-mediated lamin-A/C degradation is modulated by the phosphorylation of lamin-A/C [15]. Additionally, during the investigation of focal adhesion kinase (FAK) in cancer progression, the inhibition of FAK downregulates lamin-A/C [16]. Accordingly, the expression of lamin-A/C is modulated by kinase stimulation, such as the serine/threonine kinase Akt, the cyclin-dependent kinase Cdk, and FAK [15,16,17]. In addition, cellular environmental circumstances, such as stiffness of the matrix environment, cellular adhesion–suspension state, and mechanosensitive application, are involved in the regulation of lamin-A/C expression [17,18,19]. Matrix stiffness modulates the phosphorylated state of lamin-A/C. Reduced matrix stiffness mediates the phosphorylation of lamin-A/C at Ser22, suggesting a structural maintenance role of lamin-A/C in response to environmental status [17]. In addition, lamin-A/C is involved in cancer development and survival [20,21,22].

Histamine signaling is involved in various cancerous processes [23], although it remains debatable in lung cancer development. For example, Rovere D. et al. reported that histamine signaling is involved in lung cancer development, and reduced histamine levels have been observed in the serum of patients with lung cancer [24]. In addition, mast cell and histamine stimulation enhance cell proliferation in the alveolar adenocarcinoma cell line A549, and have had anti-cancer effects in an in vivo lung carcinoma model [25]. The application of histamine signaling in lung cancer has been carefully considered because of its biphasic role [25]. More recently, type 4 histamine receptor-associated histamine activation has been considered for the enhanced anti-cancer mechanism in T cell lymphoma [26].

The roles of lamins in cancer systems have revealed diverse aspects, and lamin-A/C expression is negatively correlated with lung cancer [27]. The stimulation of histamine receptor enhances the phosphorylation of Akt [28]. Thus, it has been speculated that histamine signaling is involved in the modulation of nuclear lamin levels and subsequent cancer processes. In this study, we find an increased expression of lamin-A/C with the enhancement of histamine signaling in lung cancer cells. The mechanism of histamine-induced lamin-A/C expression and the role of histamine signaling in the pathogenesis of lung cancer cells are determined. A regulatory mechanism that accounts for the modulation of lamin-A/C levels in cancer cells is proposed, and molecular evidence for histamine signaling in lamin-A/C is provided.

## 2. Results

### 2.1. Lamin-A/C Expression Was Attenuated by Intracellular Calcium Chelation

**Figure 1:**
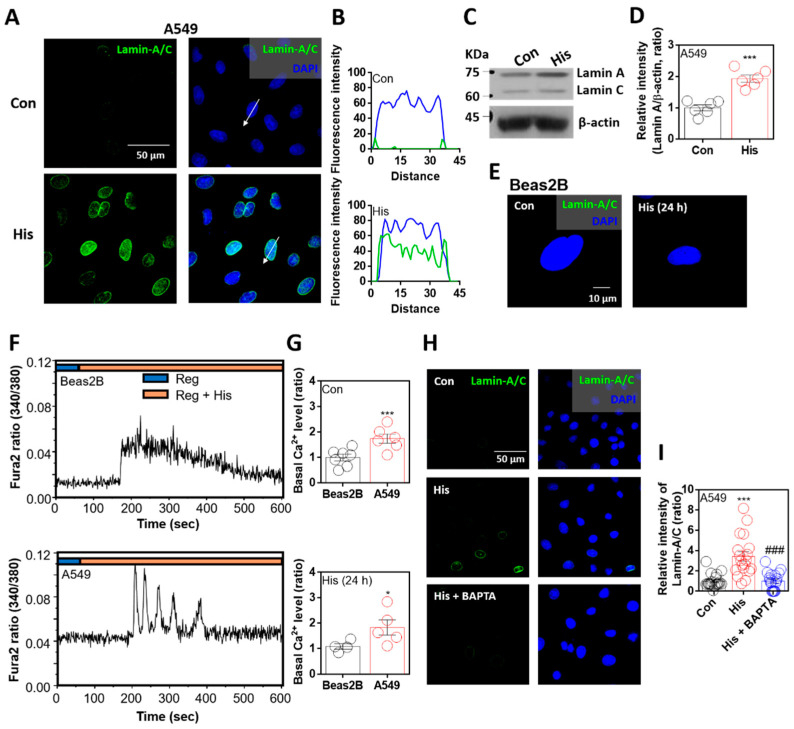
Lamin-A/C expression was attenuated by intracellular calcium chelation. (**A**) Immunofluorescence staining of lamin-A/C (green) and DAPI (blue) after histamine (His) treatment (100 μM, 24 h) in A549 cells. Scale bar represents 50 μm. Right panels were merged images. (**B**) Fluorescence intensity in the sectional distance of cells indicated by white arrows. Green and blue lines indicated lamin-A/C and DAPI, respectively. (**C**) Protein expression levels of lamin-A/C after histamine treatment (100 μM, 24 h) in A549 cells. (**D**) The graph indicates relative intensity of lamin-A. β-actin was used as a loading control. The bars represent the mean ± SEM (*n* = 6, *** *p* < 0.001). (**E**) Immunofluorescence staining of lamin-A/C (green) and DAPI (blue) after histamine treatment (100 μM, 24 h) in Beas2B cells. Scale bar represents 10 μm. Left panel was merged image. (**F**) Changes in intracellular Ca^2+^ concentration in response to histamine treatment (100 μM) in Beas2B (upper panel) and A549 (lower panel) cells. Top bars indicate the stimulated extracellular solutions. (**G**) Basal calcium levels of Beas2B and A549 cells after histamine treatment (100 μM, 24 h). The bars represent the mean ± SEM (*n* = 4~7, * *p* < 0.05, *** *p* < 0.001). (**H**) Immunofluorescence staining of lamin-A/C (green) and DAPI (blue) after histamine treatment (100 μM, 24 h) with and without BAPTA-AM (BAPTA; 10 μM, 24 h) in A549 cells. Scale bar represents 50 μm. Right panels were merged images. (**I**) The graph indicates relative intensity of lamin-A/C. The bars present the mean ± SEM (*n* = 19~20, *** *p* < 0.001 vs. control and ^###^ *p* < 0.001 vs. His-treated group). Schemes follow the same formatting.

To verify the anticancer effect of histamine on nuclear envelope integrity, cells were stimulated with histamine for 24 h. As mentioned in previous studies, reduced lamin expression is a common feature in lung cancer cells [21,27]. Immunofluorescence analysis of lamin-A/C was rarely observed in A549 lung cancer cells, whereas its expression was enhanced by histamine stimulation (Figure 1A). The sectional distance of selected cells revealed lamin-A/C intensity in randomly selected cells, and its intensity in the nuclear membrane was increased by histamine stimulation (Figure 1B). The mRNA expression of histamine receptors in A549 cells was confirmed. Levels of histamine receptor types 1 and 4 were enhanced by histamine stimulation (Supplementary Figure S1). Western blot analysis showed that the protein expression of lamin-A, not lamin-C, was enhanced by histamine stimulation (Figure 1C,D). However, the primary normal lung cell line Beas2B did not express lamin-A/C in the presence or absence of histamine stimulation (Figure 1E). It is well known that histamine stimulation induces G protein-coupled receptor activation with an increase in intracellular calcium concentration [29]. We verified that both Beas2B and A549 cells exhibited changes in calcium concentration following histamine stimulation (Figure 1F). The basal calcium levels in A549 cells with or without histamine stimulation were enhanced compared to those in Beas2B cells (Figure 1G). To test whether the enhanced intracellular calcium concentration affects lamin-A/C expression, cells were treated with calcium-chelating agent BAPTA-AM in the presence of histamine stimulation. Lamin-A/C expression was reduced in the presence of BAPTA-AM (Figure 1H,I). Interestingly, lamin-A/C expression was found to be strongly attenuated by intracellular calcium chelation.

### 2.2. Lamin-A/C Expression Was Dependent on Histamine-Mediated Signaling

**Figure 2:**
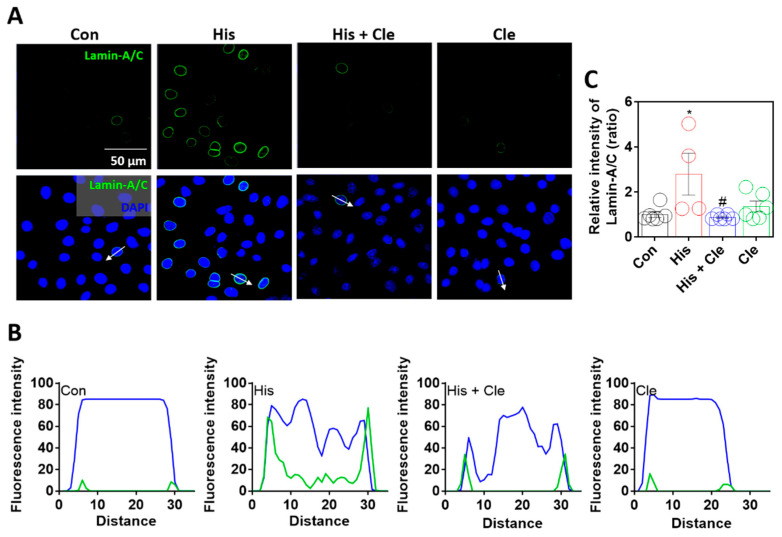
Lamin-A/C expression was dependent on histamine-mediated signaling. (**A**) Immunofluorescence staining of lamin-A/C (green) and DAPI (blue) after histamine (His) treatment (100 μM, 24 h) with or without clemastine (Cle; 10 μM, 24 h) in A549 cells. Scale bars represent 50 μm. Lower panels were merged images. (**B**) Fluorescence intensity in the sectional distance of cells indicated by white arrows. Green and blue lines indicated lamin-A/C and DAPI, respectively. (**C**) The graph indicates relative intensity of lamin-A/C. The bars present the mean ± SEM (*n* = 4~6, * *p* < 0.05 vs. control and ^#^ *p* < 0.05 vs. His-treated group).

To verify histamine-mediated lamin-A/C expression, cells were treated with the antihistamine agent clemastine chloride [30], which attenuated the histamine-mediated expression of lamin-A/C (Figure 2A–C). Sectional distance of selected cells enhanced the nuclear membranous lamin-A/C intensity by histamine stimulation, whereas co-treatment with clemastine reduced nuclear membranous lamin-A/C expression (Figure 2B). Reduced lamin-A/C expression by an antihistamine agent addressed the histamine-evoked intracellular signaling that mediates lamin biology.

### 2.3. Lamin-A/C Expression Was Dependent on Calcium/Calmodulin-Dependent Kinase II

**Figure 3:**
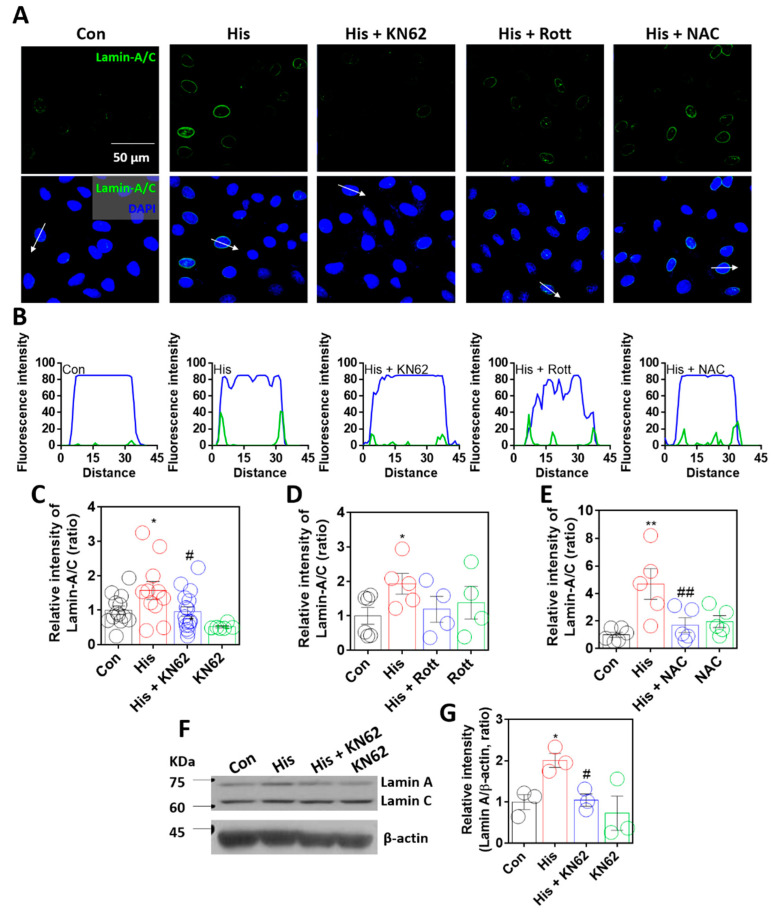
Lamin-A/C expression was dependent on Ca/CaMKII activation. (**A**) Immunofluorescence staining of lamin-A/C (green) and DAPI (blue) after histamine (His) treatment (100 μM, 24 h) with and without KN-62 (10 μM, 24 h), rottlerin (Rott; 2 μM, 24 h), or NAC (1 mM, 24 h) in A549 cells. Scale bar represents 50 μm. Lower panels were merged images. (**B**) Fluorescence intensity in the sectional distance of cells indicated by white arrows. Green and blue lines indicated lamin-A/C and DAPI, respectively. The graph indicates relative intensity of lamin-A/C after histamine treatment (100 μM, 24 h) with or without (**C**) KN-62 (10 μM, 24 h), (**D**) Rottlerin (2 μM, 24 h), or (**E**) NAC (1 mM, 24 h). The bars represent the mean ± SEM (*n* = 4~16, * *p* < 0.05 or ** < 0.01 vs. control and ^#^ *p* < 0.05 or ^##^ *p* < 0.01 vs. His-treated group). (**F**) Protein expression levels of lamin-A/C after histamine treatment (100 μM, 24 h) with or without KN-62 (10 μM, 24 h) in A549 cells. (**G**) The graph indicates relative intensity of lamin-A. The bars represent the mean ± SEM (*n* = 3, * *p* < 0.05 vs. control and ^#^ *p* < 0.05 vs. His-treated group). β-actin was used as a loading control.

It was assumed that lamin-A/C expression was dependent on increased calcium levels via histamine signaling. Thus, to determine calcium-related signaling pathways, cells were treated with inhibitors of histamine-associated signaling, such as calcium/calmodulin-dependent kinase II (Ca/CaMKII) (KN-62, [31,32], KN-93 in Supplementary Figure S2), protein kinase C (Rottlerin, [33,34]), and reactive oxygen species (ROS) (NAC, [35]). Histamine-mediated lamin-A/C expression was reduced following treatment with KN-62, KN-93, or NAC (Figure 3A–E, Supplementary Figure S2). Human non-small cell lung cancer cell line H1299 also mildly enhanced lamin-A/C expression; however, the statistical inhibitory effect of KN-62 treatment did not reveal in H1299 cells (Supplementary Figure S3). To verify the role of ROS in lamin-A/C expression, cells were stimulated with 10 μM hydrogen peroxide for 24 h. Treatment with hydrogen peroxide induced enhanced lamin-A/C expression (Supplementary Figure S4). Treatment with KN-62 or NAC revealed that the inhibition of the Ca/CaMKII-mediated signaling pathway attenuated lamin-A/C expression. Western blot analysis also confirmed that the inhibition of Ca/CaMKII attenuated histamine-mediated lamin-A expression (Figure 3F,G). A low dose of histamine at 100 nM also revealed enhanced lamin-A/C expression (Supplementary Figure S5). To verify the CaMKII-mediated signaling, cells were treated CaMKII activator oleic acid without histamine stimulation. Oleic acid also enhanced lamin-A/C expression (Supplementary Figure S6). Thus, these results indicate that lamin-A/C expression is dependent on histamine-mediated Ca/CaMKII activation.

### 2.4. Nuclear Protein Nestin Expression Was Modulated by Ca/CaMKII

**Figure 4:**
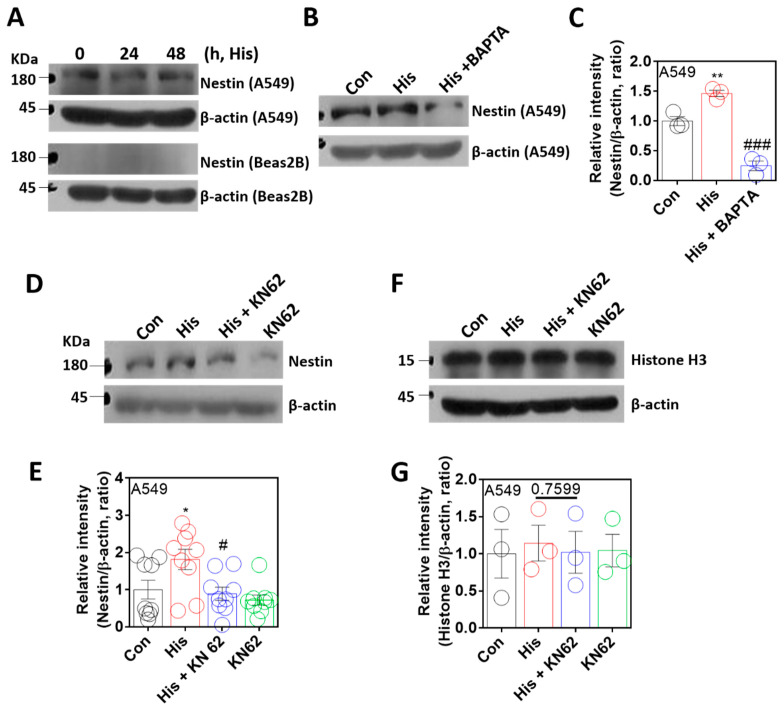
Nuclear protein nestin expression was modulated by Ca/CaMKII. (**A**) Protein expression levels of nestin after histamine (His) treatment (100 μM, 24 h and 48 h) in both A549 (upper panels) and Beas2B (lower panels) cells. β-actin was used as a loading control. (**B**) Protein expression levels of nestin after histamine treatment (100 μM, 24 h) with and without BAPTA-AM (BAPTA; 10 μM, 24 h) in A549 cells. (**C**) The graph indicates the relative intensity of nestin. β-actin was used as a loading control. The bars represent the mean ± SEM (*n* = 3, ** *p* < 0.01 vs. control and ^###^ *p* < 0.001 vs. His-treated group). (**D**) Protein expression levels of nestin after histamine treatment (100 μM, 24 h) with or without KN-62 (10 μM, 24 h) in A549 cells. (**E**) The graph indicates relative intensity of nestin. β-actin was used as a loading control. The bars represent the mean ± SEM (*n* = 9, * *p* < 0.05 vs. control and ^#^ *p* < 0.05 vs. His-treated group). (**F**) Protein expression levels of Histone H3 after histamine treatment (100 μM, 24 h) with or without KN-62 (10 μM, 24 h) in A549 cells. (**G**) The graph indicates relative intensity of Histone H3. β-actin was used as a loading control. The bars represent the mean ± SEM (*n* = 3).

The nuclear envelope protein nestin is associated with lamin-A/C, stabilizes the nuclear lamin protein, and has a cytoprotective role against oxidative stress [36,37]. To verify the role of nestin in histamine-associated lamin-A/C expression, cells were stimulated with histamine. Nestin expression was not affected by the time-dependent histamine stimulation (Figure 4A). However, BAPTA-AM treatment significantly attenuated nestin expression (Figure 4B,C). As shown by Ca/CaMKII-dependent lamin-A/C expression, nestin expression was inhibited by the treatment of KN-62 (Figure 4D,E). The protein samples, including the nuclear fraction, as shown in the nuclear marker Histone H3 [38], were also confirmed (Figure 4F,G). These results indicate that the expression of the nuclear protein nestin is modulated by histamine-mediated Ca/CaMKII activation, as shown by lamin-A/C expression.

### 2.5. Histamine-Mediated Lamin-A/C Expression Was Independent of Akt/FAK or Autophagy Signaling

**Figure 5:**
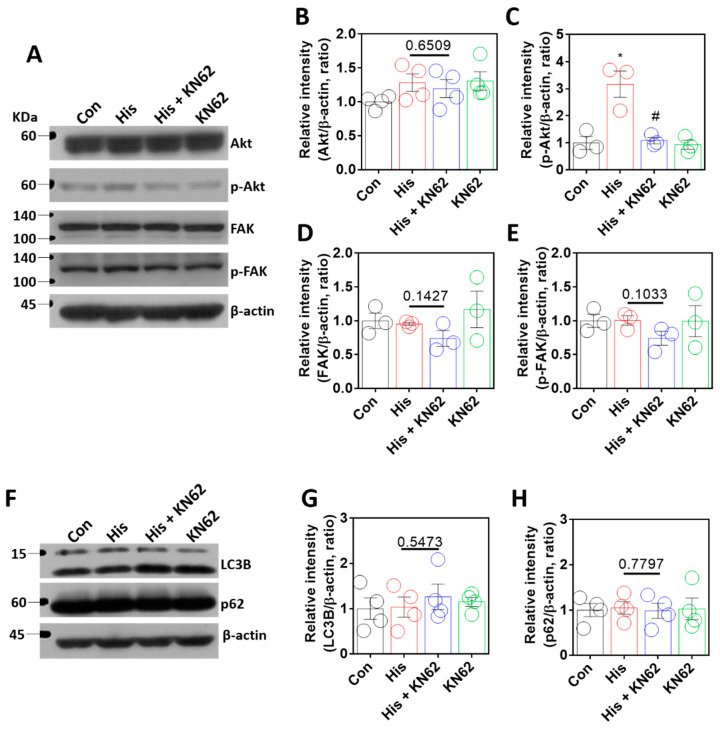
Histamine-mediated lamin-A/C expression was independent of Akt/FAK or autophagy signaling. (**A**) Protein expression levels of Akt, p-Akt, FAK, and p-FAK after histamine (His) treatment (100 μM, 24 h) with or without KN-62 (10 μM, 24 h) in A549 cells. The graph indicates relative intensity of (**B**) Akt and (**C**) p-Akt. The bars represent the mean ± SEM (*n* = 3~4, * *p* < 0.05 vs. control and ^#^ *p* < 0.05 vs. His-treated group). The graph indicates relative intensity of (**D**) FAK or (**E**) p-FAK. The bars represent the mean ± SEM (*n* = 3). (**F**) Protein expression levels of LC3B and p62 after histamine (His) treatment (100 μM, 24 h) with or without KN-62 (10 μM, 24 h) in A549 cells. The graph indicates relative intensity of (**G**) LC3B or (**H**) p62. The bars represent the mean ± SEM (*n* = 4). β-actin was used as a loading control.

The serine/threonine kinase Akt is involved in lamin-A/C expression through the regulation of lamin-A/C degradation [15] and histamine-mediated downstream signaling [39]. In addition, FAK signaling is associated with nuclear deformity in lamin-A/C expression [16]. We determined whether Akt and FAK signaling were modulated by histamine stimulation. Histamine-mediated Akt phosphorylation was inhibited by KN-62 treatment (Figure 5A–C). However, FAK expression was not affected by histamine treatment with or without KN-62 (Figure 5A,D,E). To investigate the contribution of autophagy to Ca/CaMKII-modulated lamin expression, autophagy activity was determined by the expression of autophagy markers LC3B and p62 [40,41,42]. The expression of lamin-A/C by Ca/CaMKII was not modulated by autophagic regulation (Figure 5F–H). These results indicate that histamine stimulation or the inhibition of the Ca/CaMKII pathway did not affect FAK or autophagy signaling.

### 2.6. Enhanced Lamin-A/C Expression Was Associated with Reduced Cellular Motility

**Figure 6:**
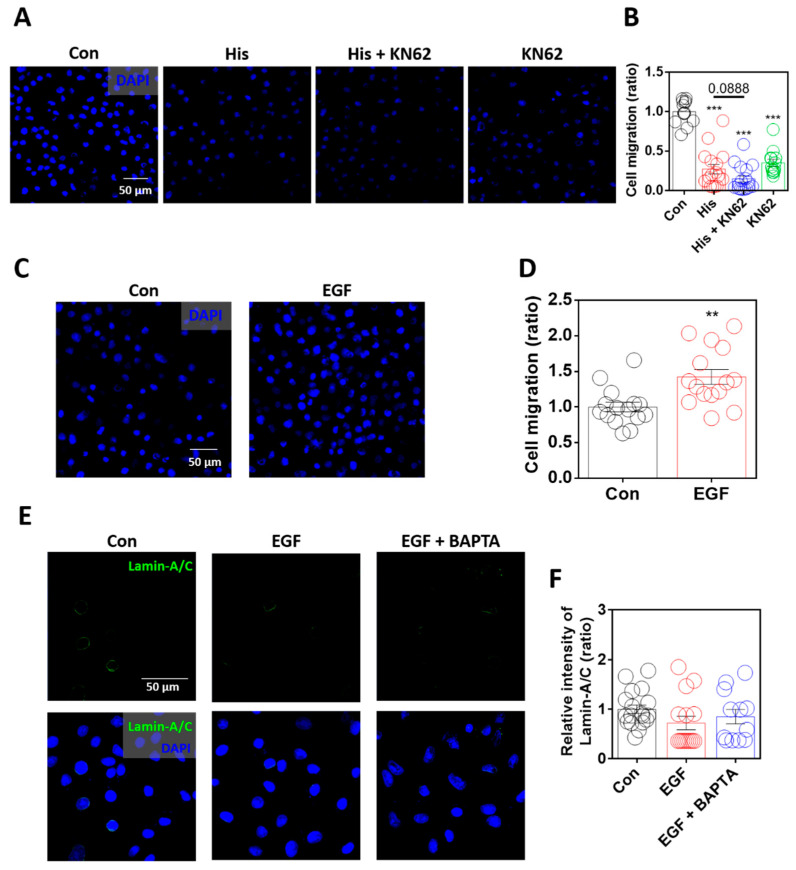
Enhanced lamin-A/C expression was associated with reduced cellular motility. (**A**) Immunofluorescence staining of DAPI (blue) after histamine (His) treatment (100 μM, 4 h) with or without KN-62 (10 μM, 4 h) in A549 cells. Scale bar represents 50 μm. (**B**) The graph indicates relative intensity of DAPI to assess A549 migration. The bars represent the mean ± SEM (*n* = 13~17, *** *p* < 0.001 vs. Control). (**C**) Immunofluorescence staining of DAPI (blue) after EGF treatment (10 ng/mL, 6 h) in A549 cells. Scale bar represents 50 μm. (**D**) The graph indicates relative intensity of DAPI to assess A549 migration. The bars represent the mean ± SEM (n = 15, ** *p* < 0.01 vs. control). (**E**) Immunofluorescence staining of lamin-A/C (green) and DAPI (blue), after EGF treatment (10 ng/mL, 24 h) with and without BAPTA-AM (BAPTA, 10 μM, 24 h) in A549 cells. Scale bar represents 50 μm. Lower panels were merged images. (**F**) The graph indicates relative intensity of lamin-A/C. The bars represent the mean ± SEM (n = 12~18).

Lamin-A/C modulates cellular migration by modulating nuclear deformity [6]. In addition, lamin loss enhances ovarian cancer cell migration [43]. To determine the role of lamin-A/C in cellular migration, the migratory ability of the cells was measured using the Boyden Transwell system. Although histamine mediates the chemotaxis of immune cells, histamine treatment dramatically attenuated cellular migration, and Ca/CaMKII inhibition also attenuated cellular migration in A549 cells (Figure 6A,B). Although inhibitors of Ca/CaMKII attenuated histamine-mediated lamin-A/C expression, the cellular migratory ability was not restored. As shown in the KN-62-only treated condition (Figure 6A,B), the global inhibition of Ca/CaMKII signaling attenuated cellular motility. The relationship between cellular migration and lamin-A/C expression in the presence of EGF was verified. Cellular migration was enhanced by stimulation with EGF (Figure 6C,D). However, the expression of lamin-A/C was not affected by treatment with EGF, even below that in the control group, similar to the presence of BAPTA-AM (Figure 6E,F). These results suggest that histamine treatment-mediated lamin-A/C expression attenuates the migration of lung cancer cells.

## 3. Discussion

This study found that a histamine-mediated intracellular calcium increase and subsequent Ca/CaMKII involvement are required for nuclear lamin-A/C expression in lung cancer cells. The results showed that histamine-mediated calcium release and the subsequent activation of Ca/CaMKII are modulatory mechanisms for lamin-A/C expression. Moreover, histamine-mediated nestin expression is associated with maintaining lamin-A/C stability.

Histamine signaling is required for the modulation of immune functions and responses through the activation of H1, H2, and H4 receptors [44], as expressed in A549 cells. The activation of the histamine receptor mediates changes in intracellular calcium concentration and ROS production [45,46]. In addition to histamine stimulation, the direct stimulation of ROS induced lamin-A/C expression. Subsequently, the enhanced calcium and ROS levels were associated with CaMKII activation. Attenuated lamin-A/C expression following treatment with a Ca/CaMKII inhibitor or ROS scavenger revealed that Ca/CaMKII signaling was associated with lamin biology. Interestingly, the nuclear envelope protein, nestin, showed a calcium/CaMKII-dependent expression pattern.

**Figure 7:**
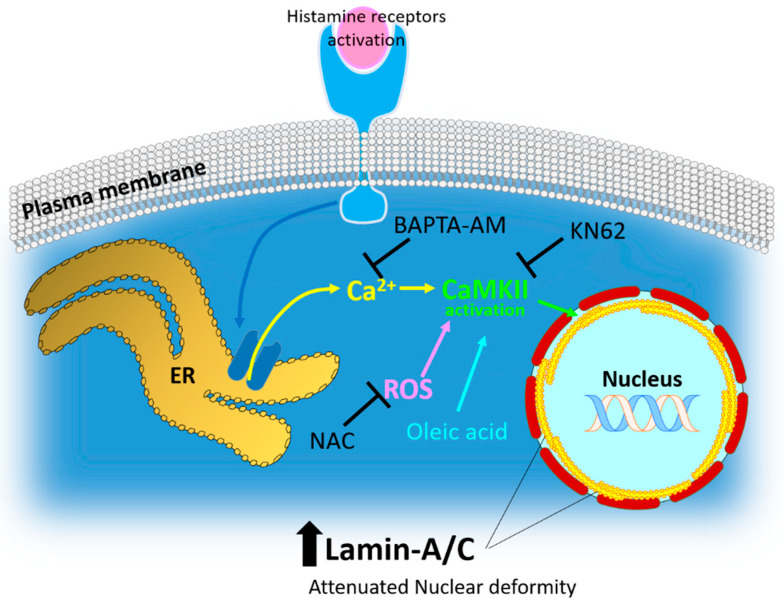
Schematic illustration of histamine-mediated intracellular signaling and several effectors on lamin-A/C expression. Histamine receptor activation induces calcium store ER-mediated calcium release and subsequent CaMKII activation. ROS stimulation and oleic acid treatment also induced lamin-A/C expression. Enhanced lamin-A/C expression involved attenuated nuclear deformity, suggesting the role of lamin biology on the histamine-mediated antitumor effects for the development of therapeutic targets for lung cancer. ROS: reactive oxygen species; NAC: N-acetyl-l-cysteine, ER: endoplasmic reticulum.

Activation of histamine receptors is known to involve cellular proliferation and migration in various cellular systems, such as fibroblasts, eosinophils, and mast cells [47,48,49]. The development and progression of various cancer types are associated with histamine receptors, particularly the H4 receptor [23,50]. In non-small cell lung cancer (NSCLC), the activation of the H4 receptor is involved in the prevention of the epithelial-to-mesenchymal transition process [51]. Agonists of the H4 receptor have been identified as potential therapeutic targets for NSCLC [44,51]. Similarly, the results revealed that the global activation of histamine receptors with histamine attenuated lung cancer cell migration (Figure 6A,B). It is speculated that histamine-induced lamin-A/C expression is involved in reduced cellular migration. However, as shown by co-treatment or a single application of KN-62 (Figure 6A,B), the global inhibition of Ca/CaMKII signaling attenuated cellular migration independent of lamin-A/C expression. Our results revealed that histamine stimulation attenuated cellular migration, whereas EGF-stimulated cells showed enhanced migration. Cells with enhanced motility upon EGF stimulation were independent of lamin-A/C levels (Figure 6E,F). The expression level of lamin-A/C in EGF-stimulated cells was similar to that in the control cells. Low levels of lamin-A/C in lung cancer cells were maintained in the presence of migratory signaling molecules, such as EGF. This could be a cellular strategy for migration through low levels of lamin-A/C expression-mediated nuclear deformation [52]. Thus, although the differential mechanism of each histamine receptor activation was not elucidated in this study, histamine stimulation including H4 receptor activation could be a promising molecular strategy through the modulation of nuclear deformation for cancer treatment (schematic illustration is represented in Figure 7).

Since lamin-A/C was first identified, its roles have been determined in various areas such as the maintenance of nuclear shape, chromatin modulation, gene regulation, cellular differentiation, development, and senescence [1,2,3,4,5,6,7,8]. The identification of the modulatory mechanism of lamin-A/C expression has been gradually developed. Lamin-A/C expression is modulated by kinases such as Akt or Cdk [15], and maintains stability in the presence of nestin [36]. The results showed that CaMKII inhibition with KN-62 mediated the decrease in nestin levels and the subsequent reduction in lamin-A/C. Therefore, the indirect effect of CaMKII inhibition on lamin-A/C expression cannot be eliminated. The findings allow for further elucidation of the molecular mechanism of Ca/CaMKII-induced lamin biology and help in the understanding of histamine-mediated antitumor effects for the development of therapeutic targets for lung cancer.

Ca/CaM signaling is associated with Akt phosphorylation. CaM inhibition attenuates Akt phosphorylation and mast cell migration [39]. In this experimental system, histamine-mediated Akt phosphorylation was inhibited CaMKII inhibition. However, activated Akt-mediated lamin-A/C degradation was not induced by histamine signaling. FAK signaling has also been associated with the modulation of lamin-A/C [16]. It is known that inhibition of FAK by PF-573228 triggers lamin-A/C degradation-mediated cellular senescence and the retardation of cellular growth in lung cancer [16]. Although their report addressed the relationship between FAK and lamin-A/C and revealed coordinated expression, the relationship seems to be independent of the linkage between FAK and CaMKII-mediated lamin-A/C expression.

Moreover, reduced and mislocalized lamin expression, including type B lamin, is considered a common feature in lung cancer [21,27,53], and the diverse roles and differential expression of lamins in cancer cells [54,55] are still challenging issues to clarify. Thus, our findings revealed that histamine signaling mediated calcium signaling and subsequent CaMKII activation, which is associated with lamin biology. Generally, Ca/CaMKII has a critical function in the modulation of cellular proliferation, invasion, and metastasis. Its inhibitors have been developed in leukemia and liver cancer [56,57]. In addition, Ca/CaMKII signaling-mediated lamin-A/C expression provides novel insight into the verification of the anti-tumor effect of histamine in lung cancer. Thus, these data establish CaMKII as a novel therapeutic target whose modulation presents new opportunities for cancer treatment. In addition to the precise modulation of CaMKII downstream signaling, activating targets of histamine signaling-mediated Ca/CaMKII may produce a beneficial effect for treating lung cancer.

## 4. Materials and Methods

### 4.1. Cell Culture

Cells of human bronchial epithelial cell line Beas2B, lung adenocarcinoma cell line A549, and the human non-small lung cell line H1299 were obtained from the American Type Culture Collection (Rockville, MD, USA). Beas2B and A549 cells were maintained in Dulbecco’s modified Eagle’s medium (#11995–065, DMEM, Invitrogen, Waltham, MA, USA) and H1299 cells were maintained in Roswell Park Memorial Institute 1640 medium (#11875–093, RPMI 1640, Invitrogen, Waltham, MA, USA) containing 10% fetal bovine serum (#16000–044, FBS; Invitrogen) and 100 U/mL penicillin–streptomycin (#15140–122, Invitrogen). The cells were cultured at 37 °C in a 5% CO_2_ and 95% air-conditioned incubator. When the cells reached 80–90% confluence, the culture medium was removed, and the cells were washed with Dulbecco’s phosphate-buffered saline (#LB001-02, DPBS, Welgene, Gyeongsan-si, Korea) and were added to new media or sub-cultured for other experiments.

### 4.2. Immunofluorescence and Confocal Imaging

Beas2B, A549, and H1299 were seeded onto cover glasses and treatment with 100 μM or 100 nM Histamine (#H7125, Sigma, Saint-Louis, MO, USA), 10 μM 1,2-Bis (2-aminophenoxy) ethane-N,N,N′,N′-tetraacetic acid tetrakis-acetoxymethyl ester (#A7076, BAPTA-AM, Sigma), 10 μM Clemastine (#SML0445, Sigma), 10 μM KN-62 (#13318, Cayman, Ann Arbor, MI, USA), 2 μM Rottlerin (#557370, Sigma), 1 mM N-acetyl-l-cysteine (#A9165, NAC, Sigma), 10 ng/mL Epidermal Growth Factor (#MAN0003587, EGF, Thermo Fisher, Waltham, MA, USA), 10 μM Hydrogen peroxide (#UN2014, H_2_O_2_, Duksan, South Korea), and 20 μM KN-93 (#13319, Cayman, Ann Arbor, MI, USA) for 24 h and 10 μM oleic acid (#01008, Sigma) for 30 min was conducted. Fixation was performed using chilled methanol (−20 °C, 10 min), and the fixed cells were added to 100 mM glycine solution for 10 min at 4 °C and washed three times with cold DPBS. To block non-specific antibody reactions, the cells were treated with 0.5% *bovine serum albumin* (BSA) in DPBS with 10% goat serum for 1 h at room temperature (22~24 °C) in darkness. The lamin-A/C antibody (#ab185014, Abcam, Cambridge, UK; 1:200 dilution factor in 0.5% BSA in DPBS with 10% goat serum) was incubated at 4 °C overnight. Following incubation, cells were washed with DPBS three times, and the cover glasses were carefully attached and mounted on glass slides with 4′,6-diamidino-2-phenylindole (DAPI)-containing Fluoromount-G^TM^ (Electron Microscopy Sciences, Hatfield, PA, USA). Fluorescence images were acquired using a Zeiss LSM700 confocal microscope (Fluoview, Carl Zeiss, Oberkochen, Germany) and analyzed with ZEN software.

### 4.3. Western Blot

Treatment of 100 μM histamine for 24 and 48 h and 10 μM BAPTA-AM, 10 μM KN-62, and 1 mM NAC for 24 h was conducted in Beas2B and A549. Cell signaling lysis buffer (containing 20 mM Tris, 150 mM NaCl, 2 mM EDTA, 1% Triton X-100, protease inhibitor mixture, and a phosphatase inhibitor mixture; Cell Signaling, Danvers, MA, USA) was added to cells, and cell membranes were disrupted by sonication. The cells were collected at 11,000× *g* for 15 min at 4 °C, and cell debris was discarded. To quantify the protein concentration, a Bradford assay (#5000001, Bio-Rad, Hercules, CA, USA) was performed. The collected cells were incubated with protein sample buffer at 37°C for 15 min to recover proteins. Extracted protein samples were loaded onto SDS-PAGE gels and then transferred onto polyvinylidene difluoride (#1620177, PVDF, BioRad) membranes soaked in methanol for activation. The membranes were incubated with 5% non-fat milk solution in TBS-T (Tris-buffered saline (TBS) and 0.5% Tween-20) for 1 h to block non-specific protein binding and then treated with lamin-A/C (#ab108595, Abcam, Cambridge, UK), Nestin (#ab6320, Abcam), Histone H3 (#4499, Cell signaling), Akt (#4691, Cell signaling), p-Akt (#9271, Cell signaling), FAK (#3285, Cell signaling), p-FAK (#3283, Cell signaling), LC3B (#NB100-2220, Novus Biologicals, Centennial, CO, USA), p62 (#ab56416, Abcam), and β-actin (#A5441, Sigma) antibodies overnight at 4 °C and washed thrice with TBS-T. β-actin was used as a loading control for Western blots. To detect the primary antibodies, the wet membranes were treated with horseradish peroxidase-conjugated anti-mouse or anti-rabbit secondary antibodies, and the protein bands on the PVDF membrane were visualized using an enhanced chemiluminescent solution (ECL, BioRad) and developed on X-ray film (Kodak, Tokyo, Japan).

### 4.4. Measurement of Intracellular Ca^2+^ Concentration

To measure the intracellular Ca^2+^ concentration ([Ca^2+^]_i_), changes in [Ca^2+^]_i_ in Beas2B and A549 cells were measured using Fura2-acetoxymethyl ester (Fura2-AM; Teflabs, Austin, TX, USA) at dual excitation wavelengths of 340 and 380 nm and an emission wavelength of 510 nm. Cells cultured on coverslips were placed in the chamber with 4 µM Fura2-AM in the presence of 0.5% Pluronic F-127 for 15 min at room temperature in darkness. Fura2 dye-loaded cells were perfused regularly for at least 4 min to stabilize the fluorescence before measuring [Ca^2+^]_i_ levels at 37°C. After perfusion with 100 µM histamine-containing regular solution, changes in [Ca^2+^]_i_ levels of cells were measured. To determine the basal [Ca^2+^]_i_ levels, cells treated with 100 µM histamine were perfused with a regular solution. The range of [Ca^2+^]_i_ at 0–50 s was defined as the basal [Ca^2+^]_i_ level. The emitted fluorescence signal was obtained with a Retiga 6000 CCD camera (Teledyne Q-Imaging, Surrey, BC, Canada) attached to an inverted microscope (Olympus, Tokyo, Japan) and analyzed using a MetaFluor system (Molecular Devices, San Jose, CA, USA). Time-lapse fluorescence images were obtained at 1 s intervals, and background fluorescent signals of images were subtracted from raw background signals at each wavelength.

### 4.5. Migration Assay

The A549 migration assay was performed using a Transwell polycarbonate membrane (8.0 μm pore size, 6.5 mm insert membrane). A549 cells (2.5 × 10^4^ cells, 200 μL) containing 1% FBS were seeded onto the permeable insert membranes. Treatment with 100 μM histamine, 10 ng/mL EGF, and 10 μM KN-62 dissolved in 500 μL of DMEM was performed on the lower plates, and then, A549 cells were incubated for 4~6 h to induce migration. The DMEM medium on the lower plates was then removed, and permeable membranes were treated with chilled methanol for 1 min at −20 °C. The methanol was removed, and the membranes were washed thrice with DPBS. The lower plate was treated with 1 μg/mL DAPI in distilled water (DW). The plates were incubated for 30 min in the dark at 37 °C in 5% CO_2_ and 95% air. Then, the DAPI solution was carefully removed, and DW was added to the lower plate at room temperature. The permeable membranes were washed twice with DW. The DAPI-incubated cells were measured at a 405 nm wavelength using a Zeiss LSM700 confocal microscope. The migration of A549 cells was determined by the number of nuclei positively stained with DAPI on the Transwell membrane.

### 4.6. MTT Assay

Beas2B and A549 cells (1 × 10^4^ and 5 × 10^3^, respectively) were seeded onto 96-well plates for 24 h and treated with 100 μM histamine for 24, 48, and 72 h and with 10 μM KN-62 for 24 h. Tetrazolium bromide dye (#298-93-1, 2 mg/mL; MTT, Merck, Burlington, MA, USA) was mixed with DPBS (37 °C), and 100 μL MTT solution was added to the cells, which were then incubated for 2 h in the dark at 37 °C in 5% CO_2_ and 95% air. The incubated supernatants were carefully removed from the plates. Dimethyl sulfoxide (100%) was added to the plates. Absorbance was measured at 570 nm using a fluorescence microplate reader (VICTOR X3, PerkinElmer, Waltham, MA, USA).

### 4.7. Total RNA Extraction and Quantitative Real Time-Polymerase Chain Reaction (RT-qPCR)

Total RNA was extracted from 100 μM histamine-treated A549 cells using RiboEx (GeneAll, Seoul, Korea). The cells were then treated with RiboEx and sonicated. Chloroform was added, and the mixture was centrifuged at 12,000× *g* for 15 min at 4 °C. The collected supernatants were analyzed according to the manufacturer’s protocol using the Hybrid-R (GeneAll) RT-PCR kit. RNA quantification was performed using the Spectrophotometer ND-1000 (Thermo Fisher Scientific), and cDNA was amplified using Accupower RocketScript Cycle RT PreMix (Bioneer, Daejeon, Korea). RT-qPCR was performed using Power Up^TM^ SYBR^TM^ Green Master Mix (#A25741, Applied Biosystems^®^, Waltham, MA, USA) and priming using the QuantStudio^TM3^ RT-PCR system (#A28567, Applied Biosystems^®^). The primers used were as follows: human GAPDH: (forward) GAC CTG ACC TGC CGT CTA GAA A, (reverse) CCT GCT TCA CCA CCT TCT TGA; human histamine receptor 1 (H1R): (forward) CAA GGT CAT GAC TGC CAT CAT C, (reverse) TGT TGT CGT ACG GCC TTG TAG A; human H2R: (forward) TCG TTC TGT GGC TGG GCT AT, (reverse) GTC TCT GTT CAG CGC AGC ATA C; and human H4R: (forward) TCA GAG AGA CGG AGG AGA AAG AGT, (reverse) CCT GGC TCT AAG CAG TTC AAC A. The RT-qPCR cycling protocol was as follows: UDG activation at 50 °C for 2 min, dual-lock DNA polymerase at 95 °C for 2 min, denaturation at 95 °C for 15 s, annealing at 55 °C for 15 s, and extension at 72 °C for 1 min.

### 4.8. Statistical Analysis

Results are presented as mean ± standard error of the mean (SEM). Statistical significance between the mean values from the two sample groups was analyzed using Student’s *t*-test. The statistical significance of the results was determined by analysis of variance in each experiment (* *p* < 0.05, ** *p* < 0.01, and *** *p* < 0.001).

## Data Availability

MDPI Research Data Policies. All data are contained within the article.

## Supplementary Materials

The supporting information can be downloaded at: https://www.mdpi.com/article/10.3390/ijms23169075/s1.
