# Supplementary material for: Lamin-A/C Is Modulated by the Involvement of Histamine-Mediated Calcium/Calmodulin-Dependent Kinase II in Lung Cancer Cells
Source: Int J Mol Sci. 2022 Aug 13;23(16):9075. doi: 10.3390/ijms23169075 (PMC9409298; doi:10.3390/ijms23169075)
Supplement: Supplementary file 1 [file ijms-23-09075-s001.zip › ijms-1855301-supplementary.pdf]

Lamin-A/C is modulated by the involvement of histamine-mediated calcium/calmodulin–dependent kinase II in lung cancer cells

Hyeong Jae Kim[1,3], Peter C.W. Lee[2,3*], and Jeong Hee Hong[1*]

Supplementary Figure S1. Kim et al.

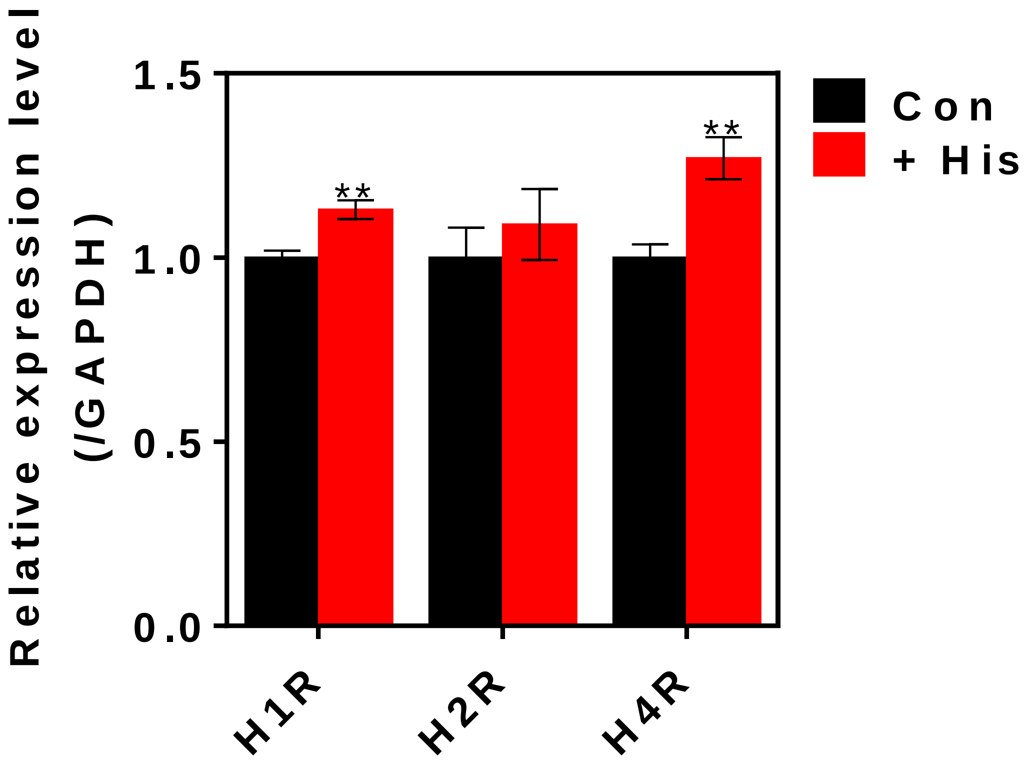

Supplementary Figure S1. The mRNA expression of histamine receptors with or without 100 μM histamine stimulation for 24 h in A549 cells. Levels of histamine receptor types 1 (H1R) and 4 (H4R), not type 2 (H2R), were enhanced by histamine stimulation.

Supplementary Figure S2. Kim et al.

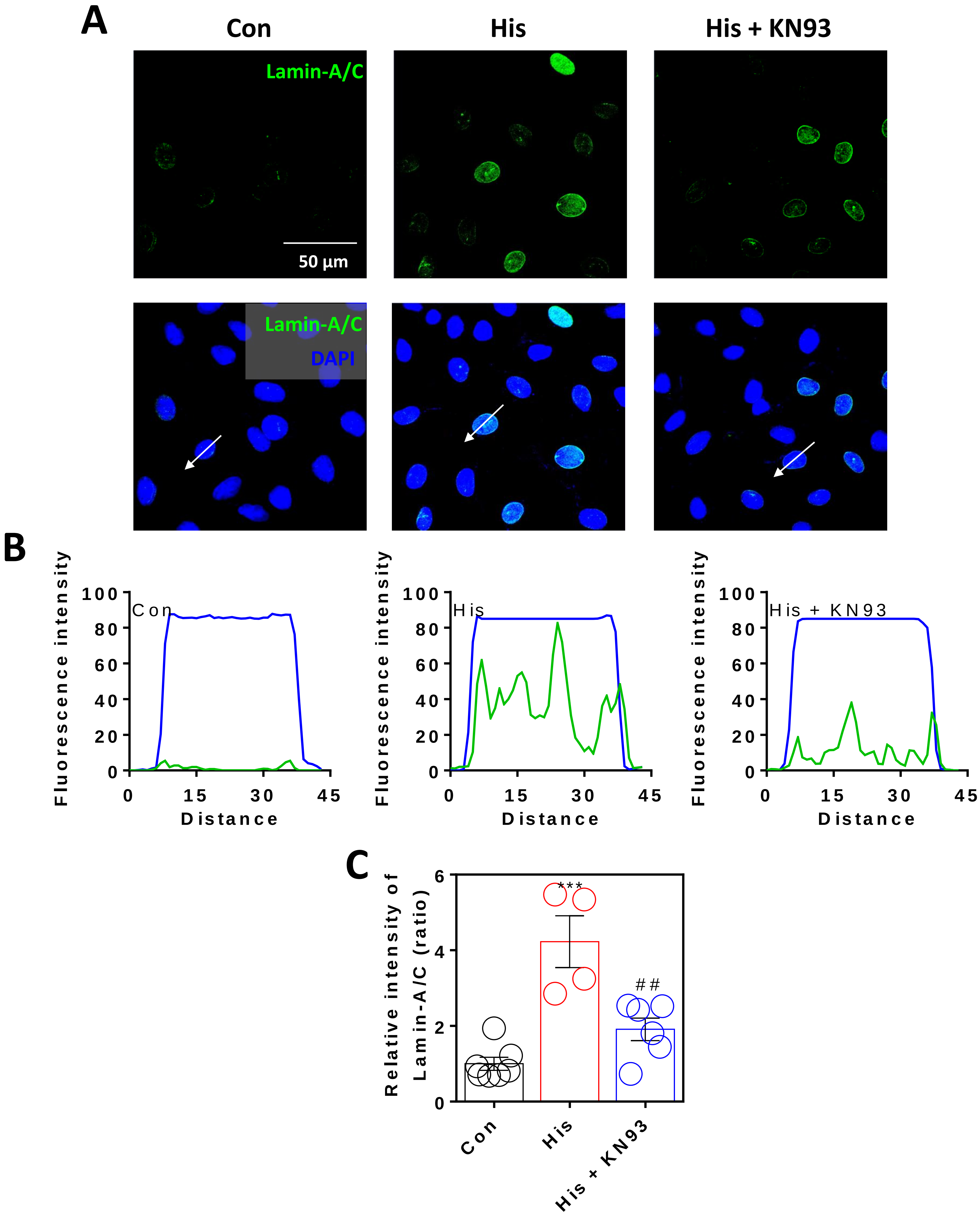

Supplementary Figure S2. Lamin-A/C expression was modulated by Ca/CaMKII inhibitor KN-93. **(A)** Immunofluorescence staining of lamin-A/C (green) and DAPI (blue) after histamine treatment (100 µM, 24 h) with or without KN-93 (20 µM, 24 h) in A549 cells. Scale bar represents 50 µm. **(B)** Fluorescence intensity in the sectional distance of cells indicated by white arrows. **(C)** The graph indicates relative intensity of lamin-A/C. The bars present the mean ± SEM (n = 4 ~ 7, ***p < 0.001 vs Control and $^{\#\#}$p < 0.01 vs His-treated group).

Supplementary Figure S3. Kim et al.

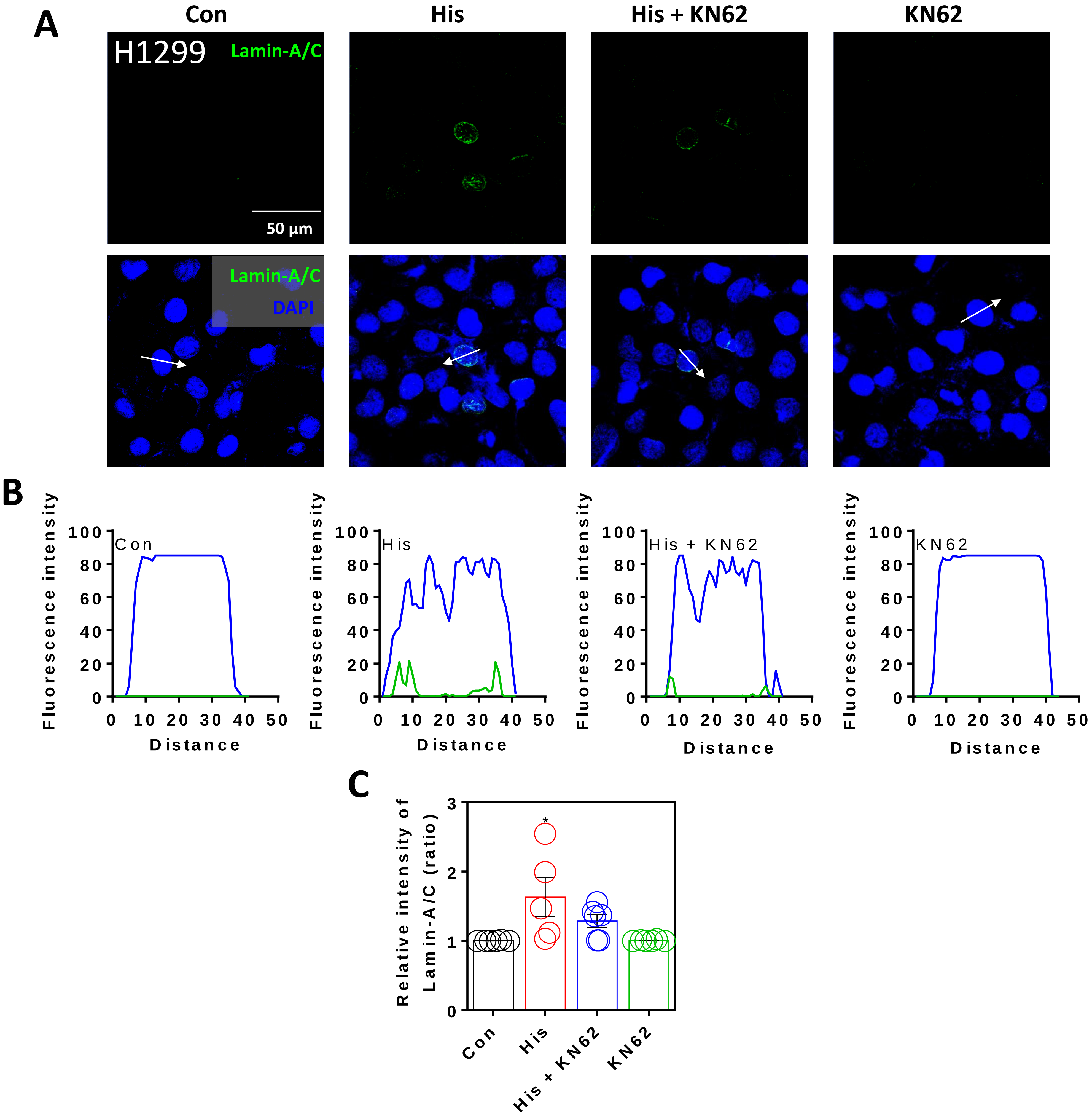

Supplementary Figure S3. Lamin-A/C expression was modulated by Ca/CaMKII in H1299 cells. **(A)** Immunofluorescence staining of lamin-A/C (green) and DAPI (blue) after histamine treatment (100 μM, 24 h) with or without KN-62 (10 μM, 24 h) in H1299 cells. Scale bar represents 50 μm. **(B)** Fluorescence intensity in the sectional distance of cells indicated by white arrows. **(C)** The graph indicates relative intensity of lamin-A/C. The bars present the mean ± SEM (n = 5 ~ 6, *p < 0.05 vs Control).

Supplementary Figure S4. Kim et al.

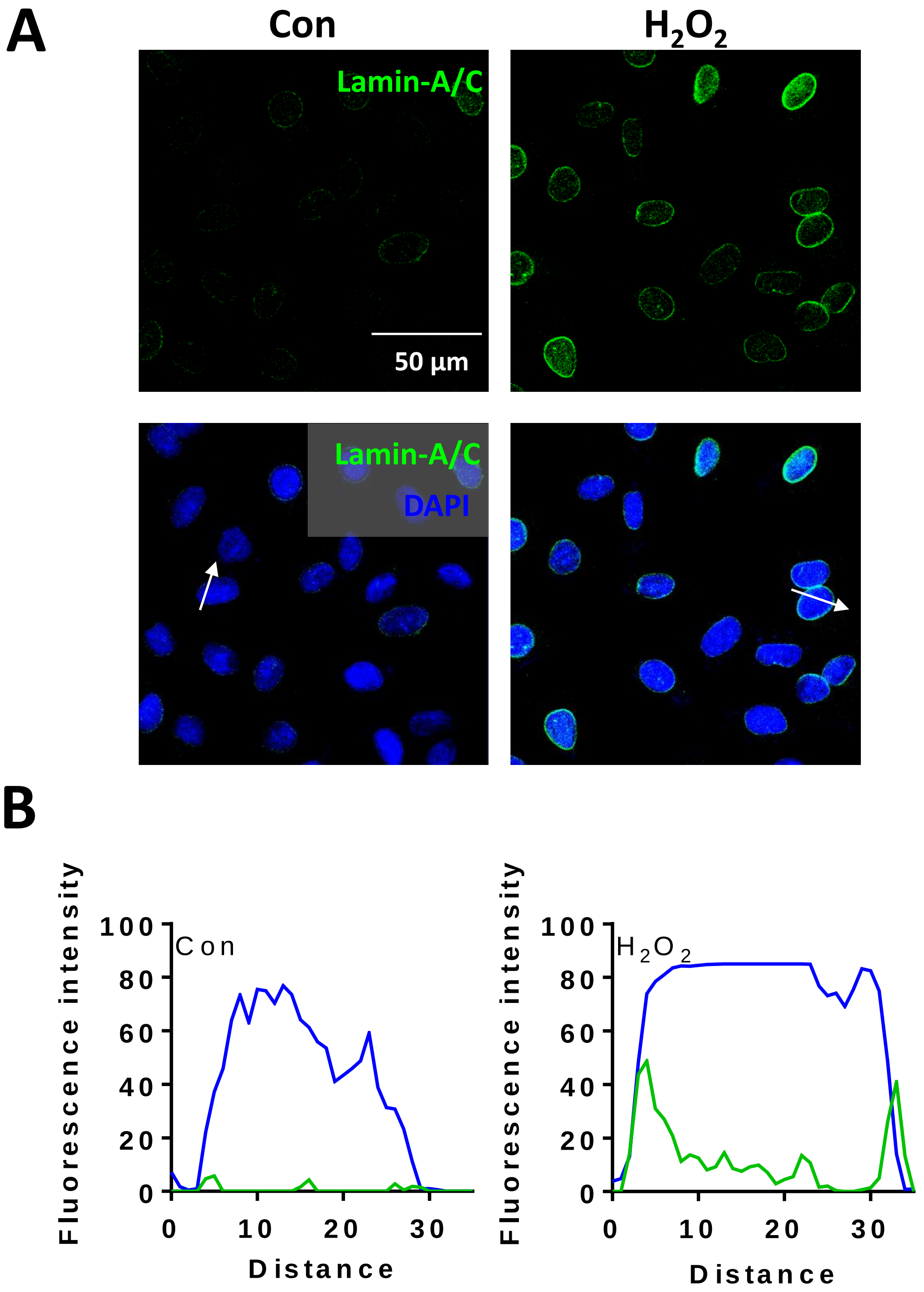

Supplementary Figure S4. Lamin-A/C expression was enhanced by ROS stimulation. **(A)** Immunofluorescence staining of lamin-A/C (green) and DAPI (blue) after H_2O_2 treatment (10 µM, 24 h) in A549 cells. Scale bar represents 50 µm. **(B)** Fluorescence intensity in the sectional distance of cells indicated by white arrows.

Supplementary Figure S5. Kim et al.

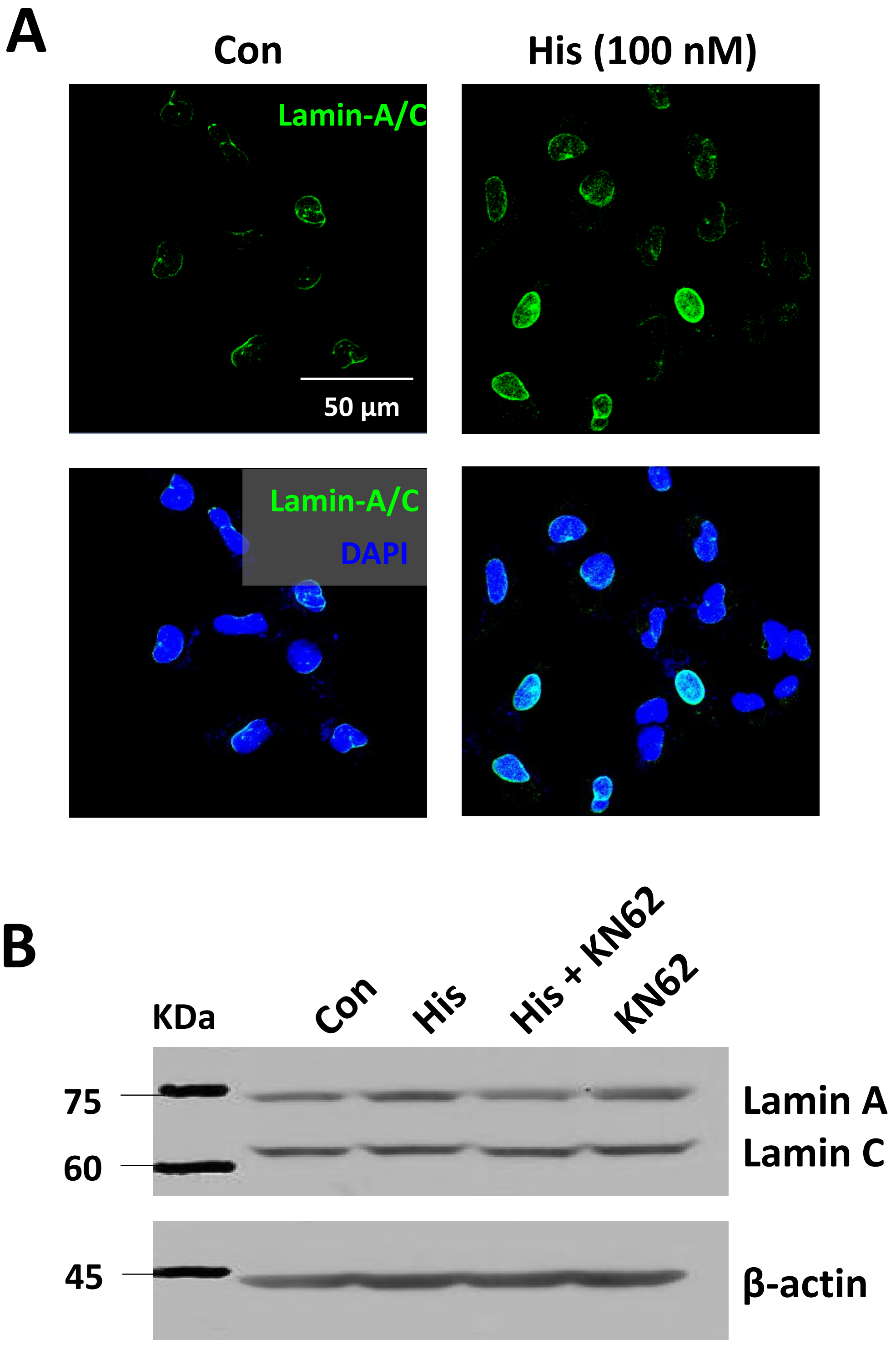

Supplementary Figure S5. Lamin-A/C expression was enhanced by low dose-histamine stimulation. **(A)** Immunofluorescence staining of lamin-A/C (green) and DAPI (blue) after histamine treatment (His, 100 nM, 24 h) in A549 cells. Scale bar represents 50 µm. **(B)** Protein expression levels of lamin-A/C after histamine treatment (100 nM, 24 h) with or without KN-62 (10 µM, 24 h) in A549 cells. β-actin was used as a loading control.

Supplementary Figure S6. Kim et al.

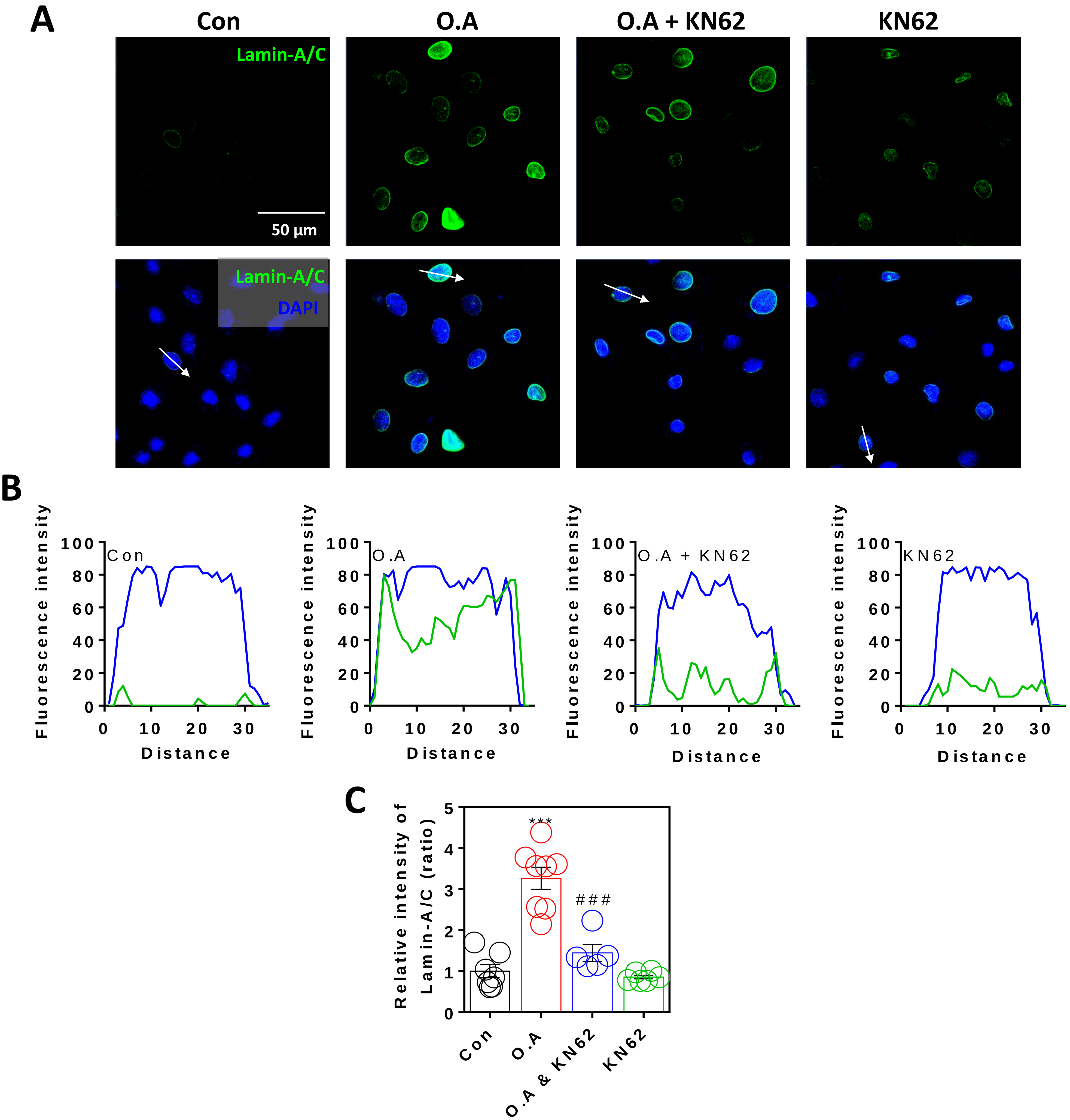

Supplementary Figure S6. Lamin-A/C expression was modulated by Ca/CaMKII activator Oleic acid. **(A)** Immunofluorescence staining of lamin-A/C (green) and DAPI (blue) after Oleic acid treatment (O.A, 10 μM, 30 min) with or without KN-62 (10 μM, 24 h) in A549 cells. Scale bar represents 50 μm. **(B)** Fluorescence intensity in the sectional distance of cells indicated by white arrows. **(C)** The graph indicates relative intensity of lamin-A/C. The bars present the mean ± SEM (n = 5 ~ 8, ***p < 0.001 vs Control and $^{###}$p < 0.001 vs His-treated group).
